# Multimeric Recombinant M2e Protein-Based ELISA: A Significant Improvement in Differentiating Avian Influenza Infected Chickens from Vaccinated Ones

**DOI:** 10.1371/journal.pone.0108420

**Published:** 2014-10-16

**Authors:** Farshid Hadifar, Jagoda Ignjatovic, Simson Tarigan, Risa Indriani, Esmaeil Ebrahimie, Noor Haliza Hasan, Andrea McWhorter, Sophie Putland, Abdulghaffar Ownagh, Farhid Hemmatzadeh

**Affiliations:** 1 School of Animal and Veterinary Sciences, The University of Adelaide, South Australia, Adelaide, Australia; 2 School of Veterinary Science, The University of Melbourne, Melbourne, Victoria, Australia; 3 Indonesian Research Centre for Veterinary Science, Bogor, West Java, Indonesia; 4 School of Molecular and Biomedical Science, The University of Adelaide, Adelaide, South Australia, Australia; 5 Faculty of Veterinary Medicine, Urmia University, Urmia, Iran; University of Georgia, United States of America

## Abstract

Killed avian influenza virus (AIV) vaccines have been used to control H5N1 infections in countries where the virus is endemic. Distinguishing vaccinated from naturally infected birds (DIVA) in such situations however, has become a major challenge. Recently, we introduced the recombinant ectodomain of the M2 protein (M2e) of H5N1 subtype as a novel tool for an ELISA based DIVA test. Despite being antigenic in natural infection the monomer form of the M2e used in ELISA had limited antigenicity and consequently poor diagnostic capability. To address this shortcoming, we evaluated the use of four tandem copies of M2e (tM2e) for increased efficiency of M2e antibody detection. The tM2e gene of H5N1 strain from Indonesia (A/Indonesia/CDC540/2006) was cloned into a pMAL- p4x expression vector and expressed in *E.coli* as a recombinant tM2e-MBP or M2e-MBP proteins. Both of these, M2e and tM2e antigens reacted with sera obtained from chickens following live H5N1 infection but not with sera from vaccinated birds. A significantly stronger M2e antibody reaction was observed with the tM2e compared to M2e antigen. Western blotting also supported the superiority of tM2e over M2e in detection of specific M2e antibodies against live H5N1 infection. Results from this study demonstrate that M2e tetramer is a better antigen than single M2e and could be more suitable for an ELISA based DIVA test.

## Introduction

Outbreaks of highly pathogenic avian influenza (HPAI) subtype H5N1 and its possible transmission to humans are of worldwide concern [1], [2]. A global spread of H5N1 began in 1997 in South East Asia ultimately spreading to Africa, Europe and the Middle East [3]. The ability of H5N1 to cause severe disease and death among bird species is related to high virus growth, particularly in tissues such as the heart and brain [4]. Moreover, transmission of H5N1 virus from infected birds to humans has been frequently reported resulting in severe disease and mortality [1], [2], [5], [6].

Control of H5N1 infections in bird populations is widely considered as an important factor for limiting human exposure to this virus [7]. Utilization of the killed avian influenza virus (AIV) as a vaccine has been widely practiced in H5N1 endemic countries. Efficient AI vaccination can reduce the amount of H5N1 shed by infected poultry into the environment and consequently exposure of naïve chickens [8]–[10]. Although vaccination can induce a broad-spectrum immunity and protection against AIV, it also has some disadvantages, including circulation and silent spread of field AIV in vaccinated flocks [11]–[13]. Vaccinated birds cannot be differentiated serologically from those naturally infected by currently available diagnostic assays such as hemagglutination inhibition (HI) test or ELISA. Therefore, differentiation between vaccinated and infected birds (DIVA) is vital to achieve effective control leading to eventual eradication of H5N1 [14], [15].

Recently, the use of the extracellular domain of the matrix protein 2 (M2e) has been suggested as an effective DIVA strategy [15], [16]. Matrix protein 2 (M2) is an structural viral protein with a significant role in virus life cycle [17]. In comparison to the hemagglutinin (HA) and neuraminidase (NA) proteins, the M2e is not subject to strong immunological selection and is relatively conserved across all subtypes of influenza A viruses [18], [19].For that reason the M2e protein has been considered as a possible candidate for development of AIV vaccines with broad-spectrum protection [9], [19].

The M2e protein is expressed on the surface of naturally infected cells in large amounts but killed AIV vaccines contain low levels of the M2e protein [12]. Consequently, the M2e antibodies are detectable only in infected but not vaccinated birds, providing the foundation of M2e based DIVA tests [12], [15], [20]. Currently M2e-DIVA are based on the use of monomeric M2e recombinant protein either as a synthetic peptide or recombinant M2e-MBP [15]. The major limitation with this test is that monomeric M2e protein is not very antigenic [21]–[23]. To address this shortcoming, increasing the number of M2e repeats was considered since theoretically, multimeric M2e could bind more antibodies than the monomeric form [22].

The aim of this study was to improve antigenicity of the recombinant M2e protein by expression of four copies of the M2e 29 amino acid long peptide, as a concatemer. This tetramer set of M2e (tM2e), which sequence originated from an Indonesian H5N1 isolate, was cloned into the pMAL-p4x expression vector and expressed tM2e protein compared to the monomeric M2e in ELISA and Western blotting.

## Materials and Methods

### Synthesis of the tM2e Gene and Cloning into the Expression Vector

For generation of tM2e protein, the open reading frame of external part of M2 protein (M2e) was selected based on multiple alignment of the M2 genes of H5N1 sequences available in the GeneBank (http://www.ncbi.nlm.nih.gov/). The selected sequence contained the first 72 nucleotides of M2 mRNA of A/Indonesia/CDC540/2006 strain (accession number EU014132.1) with amino acid sequence of “MSLLTEVETPTRNEWECKCSDSSD”. The sequence was optimized for the expression in *E. coli* since the wild-type M2e gene contained rare codons with a considerable frequency and several negatively cis-acting motifs which might hamper its expression in *E. coli*. The optimized gene was synthesized as a four tandem repeats of the above sequence and cloned into the cloning vector pMA by GENEART AG (Gewerbpark- Regensburg, Germany; www.geneart.com). The cloned gene tM2e-pMA had *Sal*I and *Bam*HI restriction endonuclease sites for cloning into pMAL-p4x expression vector (Supplementary S1).

### Expression and Purification of Recombinant tM2e-MBP Protein

TM2e-pMA cloning vector was transformed into the electro-competent DH5α strain of *E.coli* (Bioline, UK) cells according to Green and Sambrook (2012) protocol [24].After amplification, the target plasmid from the transformed cells was isolated and digested by both *Sal*I and *Bam*HI restriction enzymes (New England Biolabs, MA, USA), according to the manufacturer instructions. The digested DNA fragment was extracted using QIAquick gel extraction kit (QIAGEN, USA) and cloned into pMAL-p4x expression vector (New England Biolabs, MA, USA) containing a *mal*E gene which encodes maltose binding protein (MBP) as a carrier protein for further purification. The obtained tM2e-pMAL construct (Supplementary S1) was transformed into the DH5α *E.coli* cells with the ligating reaction and were cultured on 5 mL of 2× Yeast Tryptone (2YT) glucose broth (16 g Tryptone, 5 g NaCl, 10 g yeast extract and 0.1% D-Glucose per litre) supplemented with ampicillin (100 µg/ml).

To verify the tM2e-pMAL construct, the isolated plasmid was sequenced using vector specific primers. Positive clones from the previous steps were tested for their ability to express recombinant tM2e-MBP protein in DH5α *E.coli* cells as described previously [15] after addition of 0.3 mM isopropyl β-D-1-thiogalactopyranoside (IPTG) (Sigma, St Louis, MO, USA). tM2e-MBP protein was purified using amylose affinity resin column (New England Biolabs, Beverly, Mass., USA). The first seven fractions were collected and the protein concentration of each fraction was measured by NanoDrop spectrophometer (Thermo Scientific, DE, USA) at the wavelength of 280 nm. The fractions were then pooled, desalted and concentrated by using Vivaspin size exclusion columns with cut-off of 30 kilo Daltons (kD) (Sartorius Stedim Biotech, Germany). The purified tM2e-MBP protein was aliquoted and stored at −80°C [15].

### Analysis of purified tM2e-MBP Protein by SDS-PAGE

The purified proteins were run on SDS-PAGE with 11% concentration of acrylamide. Protein molecular weight markers (New England Biolabs.Inc, Beverly, USA) were also included and gels stained with Coomassie Brilliant Blue R250.

### Serum Samples

#### Positive Sera

M2e antibody positive sera (No = 32) were produced as described previously [15]. In brief chicks were inoculated with commercial inactivated AI vaccine (Medivac-AI, PT Medion, Bandung, Indonesia), one to three times, followed by challenge two weeks after the last vaccination with live H5N1 strain (A/chicken/West Java/Sbg-29/2007 or A/Chicken/West Java/PWT-WIJ/2006). All challenge experiments were carried out in the Biosecurity level 3 (BLS3) facilities at Indonesian Research Centre for Veterinary Science, Bogor, Indonesia. All of the positive samples (N = 32) run in tM2e ELISA test.

#### Negative sera

(a) Sera (No = 32) were obtained from chicks vaccinated in the laboratory with commercial Medivac-AI, vaccine but not challenged as described above. (b) 119 sera from commercial broilers and layers obtained from flocks in Indonesia which were vaccinated with commercial AI vaccines antibody and (c) 349 sera from non-infected and non-vaccinated field samples from commercial broiler and layer flocks in Australia which were confirmed to be AIV antibody free by an IDEXX AIV antibody test (IDEXX Laboratories, Inc). (d) One SPF chicken also served as a negative control.

### Western Blotting of M2e-MBP and tM2e-MBP proteins

Purified tM2e-MBP and tM2e-MBP, was produces for this study and both M2e-MBP (contains singe molecule of M2e co expressed with MBP) and MBP (were produced previously) were run on an 11% SDS-PAGE, transferred to a nitrocellulose membrane and blocked by immersing in 10% bovine serum albumin (BSA) in PBS for 2 hours at room temperature. After rewashing, the membranes were cut into strips and each strip incubated in a 1∶500 dilution of test sera as primary antibody for 1 hour at room temperature. Each strip was then incubated with the dilution of 1∶1000 of anti-chicken IgG conjugated to Horse-radish peroxidase (Promega, Madison, USA). After washing with PBS-T, the antigen-antibody complex was developed using diaminobenzidine (DAB) (Sigma-Aldrich Pty. Ltd) as described previously [15].

### Optimization of Recombinant M2e-MBP and tM2e-MBP ELISA

M2e-MBP, tM2e-MBP and MBP proteins, at the concentrations of 10 µg/ml were diluted in 0.1 M carbonate–bicarbonate buffer (pH 9.6) and used to coat a 96-well (150 µl/well) flat bottom microtitre plate (Sarstedt, Nümbrecht, Germany). After blocking with 5% BSA in PBS, the positive (live challenge chicken sera) and negative (vaccinated and non-vaccinated chicken sera) sera were diluted (log2 steps, starting dilution 1∶100) ELISA buffer (0.5 M NaCl, 0.1 M Tris pH 7.4, 1 mM Na2EDTA, 2% w/v BSA, 3% w/v Triton X-100, 3% w/v Tween 20), and 100 µl added to the antigen coated wells and incubated at room temperature for 1 hour. After washing, 100 µl of anti-chicken IgG conjugated to horse-radish peroxidase, diluted 1∶1000 in ELISA buffer, was added and incubated for 1 hour at room temperature. The plate was then washed and 100 µl of the substrate solution (100 µg/ml of 3,3′,5,5′-tetramethylbenzidine substrate) (Sigma, St Louis, MO, USA) added to each well and the reaction allowed to proceed for 10 minutes. The reaction was stopped by adding 50 µl of 0.16 M sulfuric acid, and optical density (OD) at 450 nm determined. Each ELISA test was repeated three times. For each serum sample, the mean OD_450_ for MBP (as carrier protein) was subtracted from the mean OD_450_ of antigen coated wells (tM2e-MBP or M2e-MBP) and the OD_450_ designated “corrected OD_450_”. Each serum sample was run in triplicate at three different times and the mean corrected OD_450_ used for statistical analysis.

### Application of tM2e ELISA on Selected Positive Serum Samples

The goal of this experiment was to evaluate the robustness of recombinant tM2e-MBP ELISA using different serum samples. Seven positive sera (Table 1) at the dilution of 1/200 were used to compare the performance of tM2e versus M2e at the same dilution of coating antigen (10 µg/ml) in ELISA. As described in Table 1, the differences between sera used were the number of vaccination before challenge, The test were ran three times for each sample and mean corrected ODs of each serum were used for comparison between different test groups.

**Table 1:** Positive serum samples used in the study*.

Name of Sera	1 time vaccinated	2 times vaccinated	3 Time vaccinated
D10^a^	√	-	-
B47^a^	√	√	-
B38^a^	√	√	-
B1^a^	√	√	-
B2^a^	√	√	-
PL80^b^	√	√	-
A17^a^	√	√	√

* Birds were immunized once (D10), twice (B47, B38, B1, B2, PL80) or three times (A17) with a commercial H5N1 vaccine, at four weekly intervals. Two weeks after the last immunization, birds were challenged with H5N1 strains ^a^ A/chicken/West Java/Sbg-29/2007 or ^b^ A/Chicken/West Java/PWT-WIJ/2006. Two weeks after the challenge, sera were collected for assay in haemagglutination inhibition test and M2e and tM2e ELISA.

### Evaluation of tM2e-ELISA field serum samples

In order to evaluate the application of tM2e-MBP ELISA as a DIVA test in field condition 532 serum samples (described above) were tested in an indirect ELISA. Test sera were diluted 1∶200 in dilution buffer (DB) and run in duplicate in tM2e-MBP ELISA test. Positive and negative controls were included in all assays in quadruplicate. The test ran at the same condition described at the previous step. Corrected ODs (450 nm) of each serum in recombinant tM2e ELISA were recorded individually and compared between different test groups. ANOVA and mean comparison using Tukey's test was used to evaluate the ability of tM2e-based ELISA as a DIVA test to distinguish infected sera from the vaccinated and non-vaccinated sera. The cut off value for field serum samples were calculated using the two-graph receiver operating characteristic (TG-ROC) analysis [25].

### Statistical Analysis

Two-way analysis of variance (ANOVA) was used to compare the OD_450_ obtained in ELISA with recombinant tM2e-MBP and M2e-MBP proteins and M2e antibody positive and negative sera. Mean comparison was also carried out by Tukey's test using MINITAB 16 package (www.minitab.com) (Table 2). The positive and negative sera (as detailed above) were assumed as treatments (test groups). The serum dilutions were considered as replications (blocks).

**Table 2:** ANOVA and mean comparison by Tukey's method of ELISA OD values of positive, SPF and sera from vaccinated chicks.

Mean Comparison				
Test group	Number*	Mean	SE Mean	Grouping
tM2e+Challenged	9	1.46	0.186	A
M2e+Challenged	9	0.62	0.173	B
tM2e+Vaccinated	9	0.06	0.029	C
M2e+Vaccinated	9	−0.13	0.065	C
tM2e+SPF	9	0	0.002	C
M2e+SPF	9	0	0.001	C

Two Way ANOVA			
Source of Variation	Degree of Freedom	F-value	P-value
Test group	5	33.98	0.00
Dilution	8	1.27	0.28
Error			
R-Square = 75.91			

* From each positive, vaccinated and SPF groups, three samples were tested in triplicate in both tM2e-MBP and M2e-MBP ELISAs.

Regression modeling of ELISA OD_450_ with different dilutions of M2e-MBP and tM2e-MBP was performed using Microsoft Office Excel 2010. Model selection was carried out based on R-square statistics. For applicability experiment, T test (MINITAB 16 package) was used to compare the OD_450_ of tM2e-MBP versus M2e-MBP with different positive serum samples. Analysis of variance and mean comparison using Tukey's test were used to compare the effects of infected, negative and vaccinated field sera based on corrected OD_450_.

### Ethics statement

All animal work was performed at the Indonesian Research Centre for Veterinary Science, Bogor, Indonesia and experimental procedures were approved by its Research Committee.

The experimental chickens were managed by a veterinarian who specializes in animal studies based on the guidelines of the National Health and Medical Research Council of Australia. The birds were monitored daily for clinical signs, morbidity, and mortality. All birds were bled via brachial vein and by cardiac puncture at the terminal step just after CO_2_ euthanasia.

## Results

### Characterization of Recombinant tM2e-MBP by SDS-PAGE and Western Blotting

**Figure 1:**
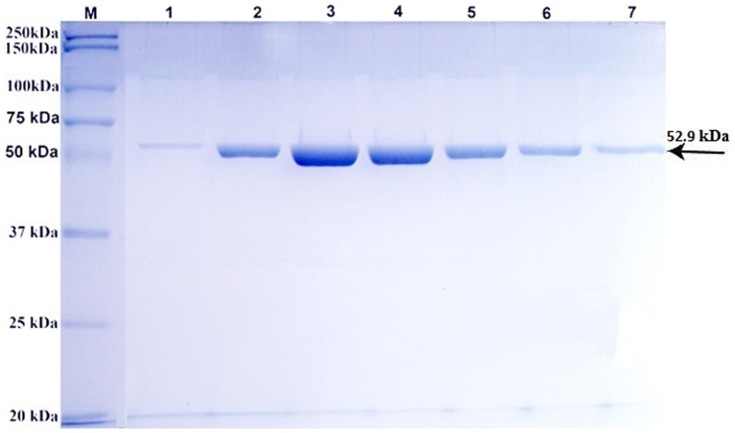
SDS-PAGE analysis of purified tM2e-MBP recombinant protein. Lane M: Molecular weight markers. Lanes 1 to 7: Fractions of purified recombinant tM2e-MBP protein with molecular weight of 52.9 kD (arrow).

tM2e-MBP protein was expressed in DH5α strain of *E.coli* and subsequently purified. SDS-PAGE analysis of seven different fraction of the purified tM2e-MBP protein showed a band of molecular weight of 52.9 kD that included 42.5 kD of MBP and 10.4 kD of tM2e protein (molecular weight of each M2e monomer was 2.6 kD) (Figure 1).

**Figure 2:**
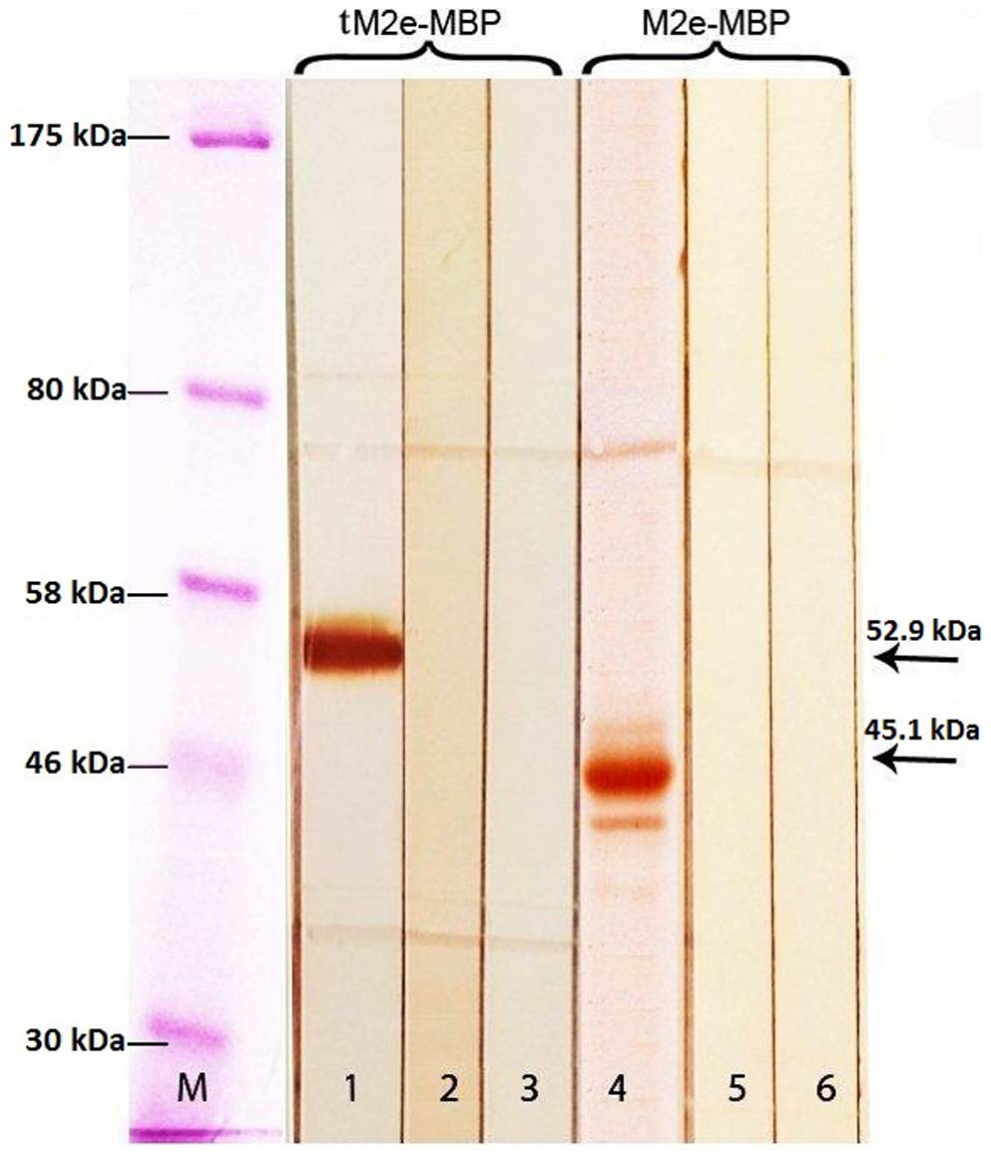
Western blotting of recombinant tM2e-MBP and M2e-MBP proteins with positive, SPF and sera from vaccinated chicks. Lane M: Molecular weight markers. Lane 1–3: Reaction of tM2-MBP with positive serum (live virus challenge), SPF, and sera from vaccinated chicks, respectively. Lane 4–6: Reaction of M2e-MBP with positive serum, SPF and sera from vaccinated chicks, respectively.

Western blotting showed that tM2e-MBP reacted with positive (live challenge) sera in the same manner as M2e-MBP, which was included as a control. Negative sera from SPF and vaccinated chicks did not react with either tM2e-MBP or M2e-MBP antigens (Figure 2). This result indicated that the tM2e-MBP was antigenic and recognized by M2e antibodies and therefore could be suitable for developing a DIVA test.

### Recombinant tM2e-MBP is better DIVA antigen than monomeric M2e-MBP

Western blotting indicated that tM2e-MBP and M2e-MBP were antigenic. Both antigens were compared in ELISA and reacted with positive sera, but improved reactivity was observed with tM2e as compared to M2e at the same protein concentration (Figure 2).

**Figure 3:**
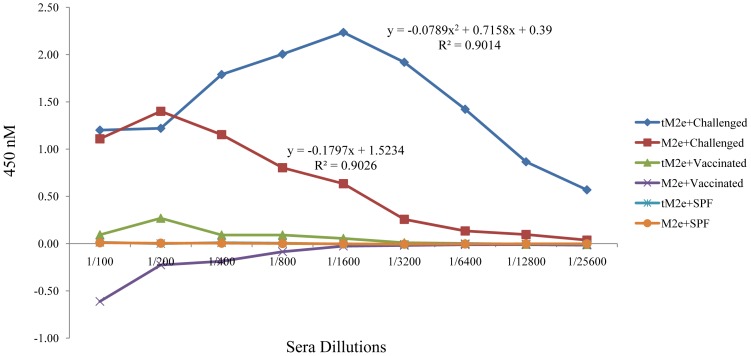
Reaction of tM2e-MBP and M2e-MBP recombinant proteins with positive, vaccinated and SPF sera in ELISA. Each serum sample run in triplicate at three different times and the mean corrected OD is presented as error bar.

**Figure 4:**
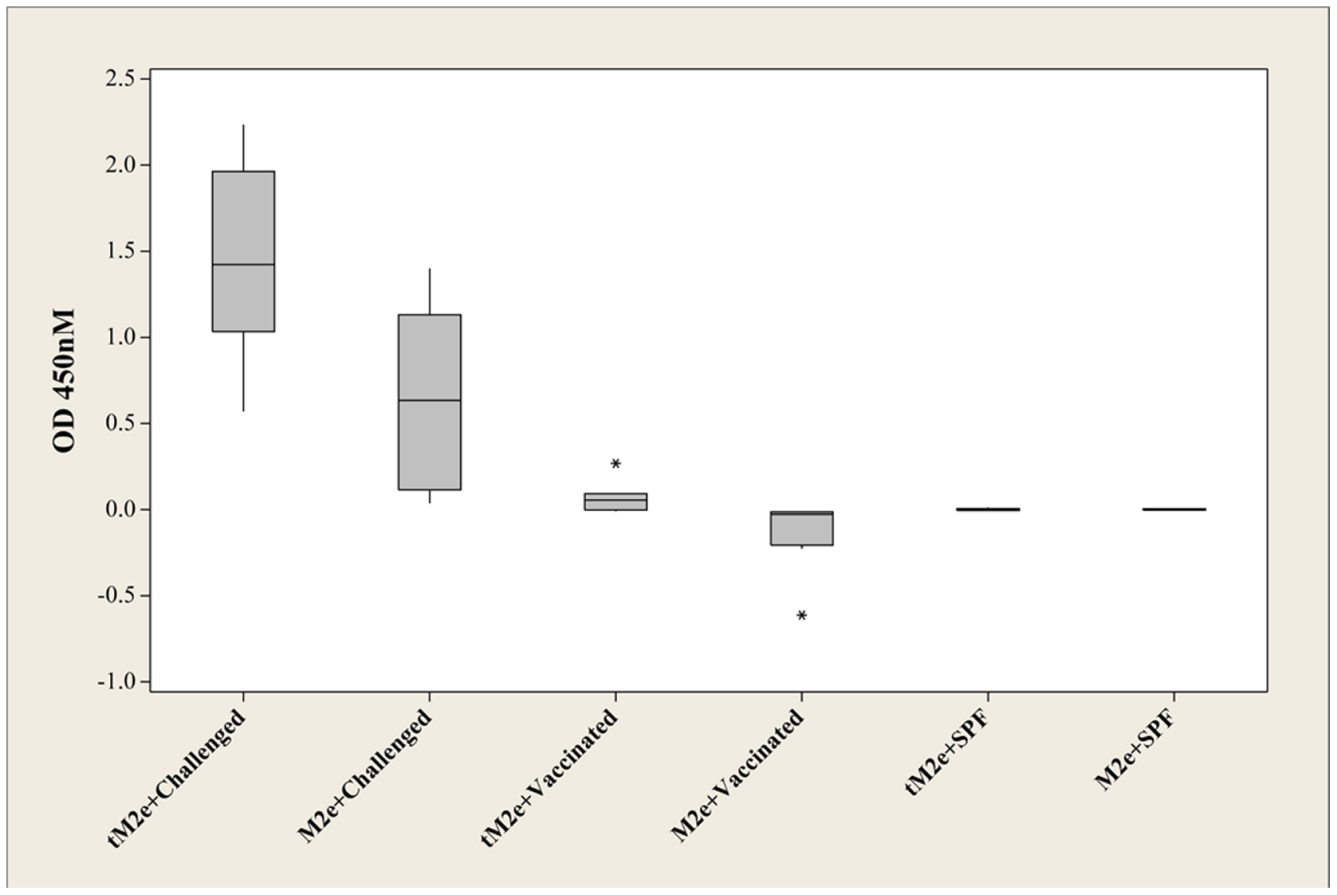
Boxplot of ODs obtained with tM2e-MBP and M2e-MBP with positive, vaccinated and SPF sera showed in **table 2**. The asterisks represent small number of the cases which are far from common ranges of plus and minus standard deviation (±SD).

In a titration ELISA, the reactivity of tM2e-MBP and M2e-MBP was compared against positive, SPF and vaccinated sera (Figure 3). It has been reported previously that some serum samples from both infected and vaccinated birds may contain some anti-MBP antibodies [15]. MBP is a carrier protein for both tM2e-MBP and M2e-MBP antigens, therefore any background caused by cross reactivity of anti-MBP antibodies to the test samples needed to be subtracted. We removed the MBP background by subtracting the OD of MBP from the OD of each individual serum. Corrected OD are presented in Figure 3. Positive serum reacted strongly with tM2e-MBP and M2e-MBP proteins, while sera from vaccinated and SPF birds did not react (Figure 3). Notably, reaction of positive serum to the tM2e-MBP protein was significantly higher in comparison to the reaction with the M2e-MBP (Figures 3 and 4). Regression analysis of the reactivity of tM2e-MBP antigen with positive serum in ELISA followed a polynomial (power 2) regression model (R-square = 90.14%) indicating the strong reactivity of tM2e even at low antigen concentrations. In contrast, M2e-MBP produced a linear trend with positive serum (R-square = 90.26%), with a sharp decrease in reactivity at lower concentrations.

The superiority of tM2e-MBP over M2e-MBP as a DIVA antigen is apparent in the dilution response (Figure 3). The reactivity of M2e-MBP with positive serum (challenged) started to decrease at the 1/200 dilution with the endpoint titer of 1/3200. In contrast, this loss in reactivity was only visible with tM2e-MBP at dilutions of sera of 1/1600 and the end point titer was at 1/25,600. In other words, the tM2e-MBP can efficiently detect M2e antibodies when present at the low concentration. ANOVA analysis confirmed that the difference between tM2e-MBP and M2e-MBP proteins in reactions with M2e positive sera were statistically significant (p = 0.05). Mean comparison by Tukey's method also showed that tM2e-MBP antigen has significantly higher OD (p = 0.01) with positive serum in ELIS compared to monomeric M2e antigen (Table 2).

Optical densities obtained from the ELISA of tM2e-MBP and M2e-MBP against a range of positive sera (listed at Table 1) and negative sera are presented as boxplot in Figure 4. It shows high reactivity of recombinant proteins particularly tM2e-MBP in distinguishing sera from live H5N1 infection from vaccinated and negative (not vaccinated not infected) sera.

### Comparison of Reactivity Response of tM2e-MBP and M2e-MBP on Selected Positive and Negative Serum Samples

**Figure 5:**
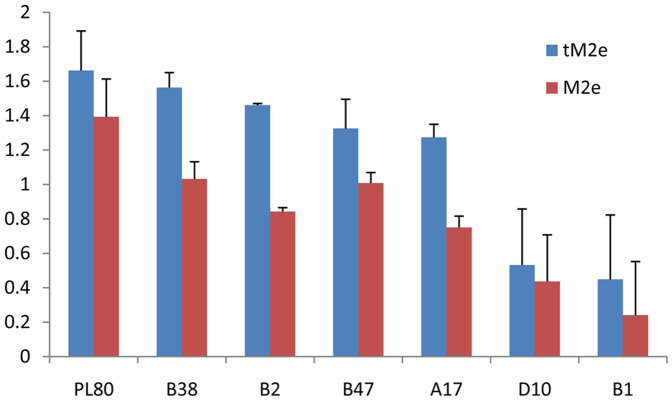
Reaction of tM2e-MBP and M2e-MBP proteins with positive sera in ELISA. The positive sera are described in Table 1. Pared T test highlighted the statistically higher reactivity (p = 0.05) of tM2e-MBP compared to M2e-MBP.

Using an ELISA test, we evaluated the reactivity of tM2e-MBP and M2e-MBP proteins with a range of positive sera (PL80, B38, B2, B47, A17, D10 and B1). As indicated in Figure 5, all seven sera produced significantly higher OD with the tM2e-MBP compared to M2e-MBP. OD of M2e-MBP ranged from 0.24 (in B1 serum) to 1.40 (in PL80). In contrast for tM2e-MBP, OD ranged from 0.45 (B1) to 1.67 (PL80). Pared T test highlighted the statistically higher reactivity (p = 0.05) of tM2e-MBP compared to M2e-MBP.

### Evaluation of field sera in tM2e-MBP ELISA

**Figure 6:**
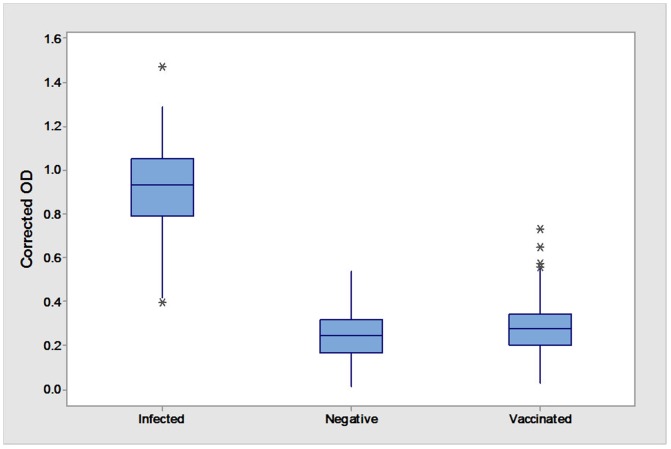
Two-graph receiver operating curve (TG-ROC) analysis of tM2e-MBP ELISA OD for field (vaccinated or non-vaccinated) and challenged (positive) sera. The cut-off value was chosen to be at 95% sensitivity and 95% specificity.

The tM2e-MBP ELISA as a DIVA test was evaluated using three groups of sera, challenged (positive), vaccinated and non-vaccinated birds. All 32 positive serum samples were positive in the tM2e-MBP ELISA (OD ranged from 0.396 to 1.471 mean). Of 151 vaccinated sera, 6 were positive with OD values ranging from 0.410 to 0.649 (specificity 96.15%). Two out of 349 sera of non-vaccinated and non-infected sera were positive with the OD values of 0.422 and 0.537 (specificity 99.43%) (Figure 6).

**Figure 7:**
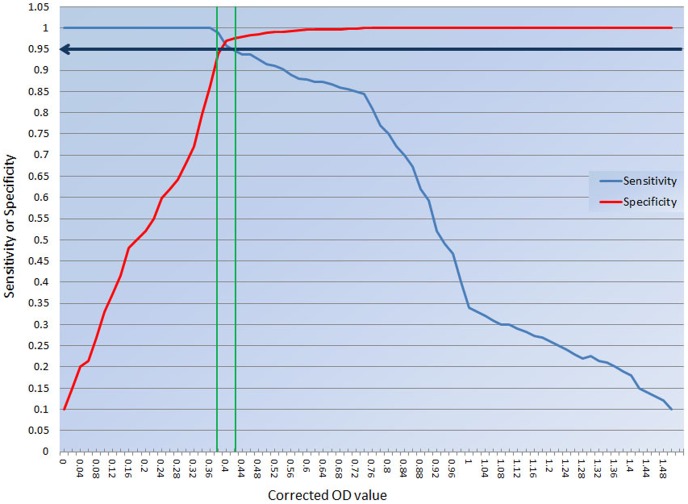
Evaluation of tM2e-MBP ELISA using sera from infected (challenged), non-vaccinated, and vaccinated chickens. ANOVA and mean comparison using Tukey's test highlighted significant results in samples from infected birds compared to negative and vaccinated birds at p = 0.01. ANOVA and Tukey's tests showed statistically higher (p = 0.01) reactivity of infected (positive) compared to vaccinated and (negative) sera.

Based on the two-graph receiver operating curve (TG-ROC) analysis, the cut off values were calculated as an intermediate range between 0.390 and 0.430, corresponding to 95% sensitivity and specificity (Figure 7). These results demonstrate high applicability of tM2e-MBP ELISA as DIVA test in field samples with the sensitivity of 100% and the specificity of (98.43%) in all tested samples. ANOVA and Tukey's tests showed statistically higher (p = 0.01) reactivity of infected (positive) compared to vaccinated and (negative) sera.

## Discussion

Vaccination is one of the effective measures for the control of HPAI in poultry in endemic countries. The important component of this approach is a need to distinguish between vaccinated and naturally infected poultry. Recently, two M2e based ELISAs have been described for possible use as DIVA test to fulfil this need [10], [15].

In this study, we attempted to increase the efficiency of the previously described M2e ELISA by using the four tandem repeats of the M2e (tM2e) instead of single M2e peptide, as an antigen. Sequence analysis based on hydrophobicity plotting and multiple alignment of M2e genes of available H5N1 sequences showed that the selected sequence (A/Indonesia/CDC540/2006, H5N1) had higher levels of homology with circulating strains in poultry farms in Indonesia and also possesses the greatest homology to the selected strains for the challenge experiment. Additionally, the selected peptide for expression has the highest hydrophobicity and stronger antigenicity.

Using tM2e as a recombinant protein with MBP, a higher signal and lower background was obtained than when M2e-MBP was used at the same concentration. This is likely because of a higher copy number of M2e present in the coated protein and consequently higher binding capacity for any M2e-specific antibodies. Also with the tM2e-MBP antigen there was less variability in the background OD obtained with vaccinated sera, increasing the test specificity.

Compared to the previous study [15], the tM2e-MBP ELISA test shows higher sensitivity and specificity to discriminate M2e antibodies in sera of infected birds from vaccinated or non-vaccinated birds. On the other hand, two-graph receiver operating curve (TG-ROC) analysis on field and challenged serum samples provided a suitable range of cut off points for obtaining better performance when we expect maximum sensitivity for the test.

We believe that the tM2e-MBP ELISA could be a useful DIVA test in sero-monitoring of poultry farms that practice vaccination in regions where H5N1 is endemic. The larger size of tM2e-MBP protein provides another opportunity for enzymatic digestion of the recombinant protein followed by double purification of the target peptide increases the purity of the antigen and decreases non-specific reactions in DIVA-ELISA tests. This enhanced sensitivity of the tM2e DIVA-ELISA test shows higher applicability than M2e when tested against a range of positive and vaccinated sera.

## Supporting Information

Checklist S1 ARRIVE Checklist.
(PDF)

Supplementary S1 pMAL-tM2e expression vector (Generated by “SnapGene viewer”, 2013).
(DOCX)
