# Supplementary figures and images for: Multimeric Recombinant M2e Protein-Based ELISA: A Significant Improvement in Differentiating Avian Influenza Infected Chickens from Vaccinated Ones
Source: PLoS One. 2014 Oct 16;9(10):e108420. doi: 10.1371/journal.pone.0108420 (PMC4199618; doi:10.1371/journal.pone.0108420)

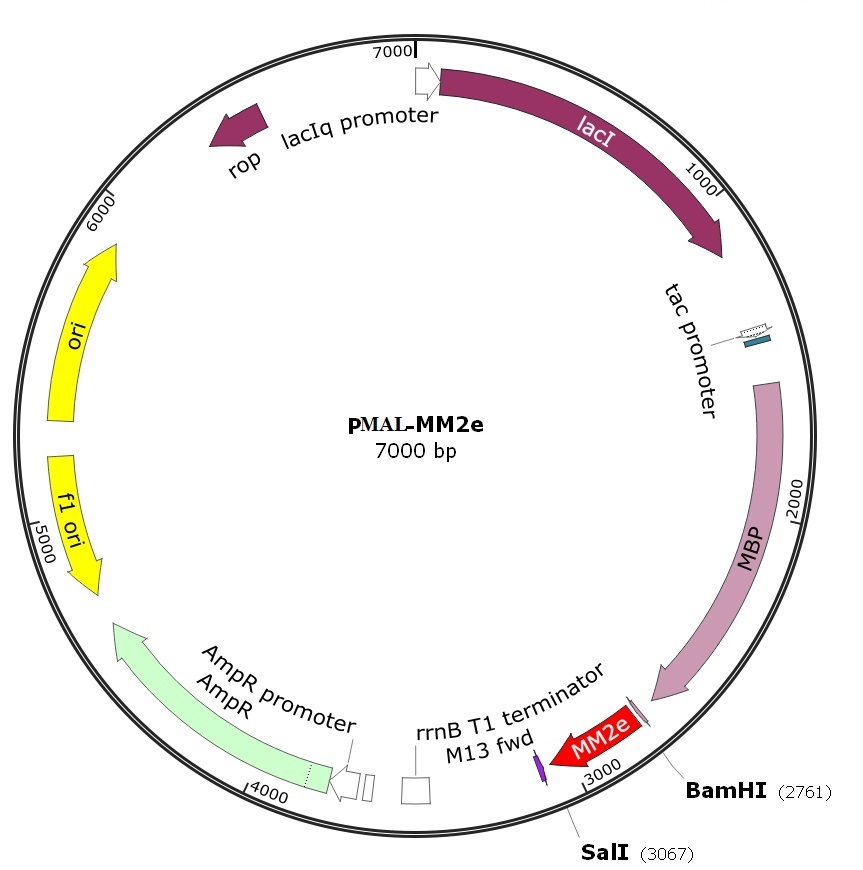


**Supplementary S1.** pMAL-tM2e expression vector (Generated by “SnapGene viewer”, 2013)

Supplement: Supplementary S1 — pMAL-tM2e expression vector (Generated by “SnapGene viewer”, 2013). (DOCX) [file pone.0108420.s002.docx]
